# Object-based visual working memory: an object benefit for equidistant memory items presented within simple contours

**DOI:** 10.1007/s00426-022-01757-w

**Published:** 2022-10-29

**Authors:** Gülşen Balta, Güven Kandemir, Elkan G. Akyürek

**Affiliations:** 1 Department of Psychology, Experimental Psychology, University of Groningen, Grote Kruisstraat 2/1, 9712 TS Groningen, The Netherlands; 2 Research School of Behavioural and Cognitive Neurosciences, University of Groningen, Groningen, The Netherlands

## Abstract

Previous research has shown that more information can be stored in visual working memory (VWM) when multiple items belong to the same object. Here, in four experiments, we investigated the object effect on memory for spatially equidistant features by manipulating simple, task-irrelevant contours that combined these features. In Experiments 1, 3, and, 4, three grating orientations, and in Experiment 2, one color and two orientations, were presented simultaneously to be memorized. Mixture modeling was applied to estimate both the precision and the guess rates of recall errors. Overall results showed that two target features were remembered more accurately when both were part of the same object. Further analysis showed that the probability of recall increased in particular when both features were extracted from the same object. In Experiment 2, we found that the object effect was greater for features from orthogonal dimensions, but this came at the cost of lower memory precision. In Experiment 3, when we kept the locations of the features perfectly consistent over trials so that the participants could attend to these locations rather than the contour, we still found object benefits. Finally, in Experiment 4 when we manipulated the temporal order of the object and the memory features presentations, it was confirmed that the object benefit is unlikely to stem from the strategical usage of object information. These results suggested that the object benefit arises automatically, likely at an early perceptual level.

**Supplementary Information:**

The online version contains supplementary material available at 10.1007/s00426-022-01757-w.

## Introduction

Working memory (WM) has been defined as a system that maintains information for a brief period to be used for various cognitive functions (Baddeley, 2003). The strongly limited capacity of WM in particular has attracted the attention of researchers (Brady et al., 2011). WM capacity is an important topic, considering that it is highly correlated with several major cognitive abilities, such as reading comprehension, fluid intelligence and executive function (Daneman & Carpenter, 1980; Fukuda et al., 2010; Miyake et al., 2001). Estimates of WM capacity range from just four to seven items (Cowan, 2001, 2005). Fortunately, this seems less than it is, as items can consist of combinations of multiple individual elements, referred to as chunks (Miller, 1956). Chunking can dramatically increase the amount of information maintained in memory (Chen & Cowan, 2005; Gobet et al., 2001; Miller, 1956).

A compelling demonstration of chunking information into visual objects was given by Luck and Vogel (1997). These authors measured visual WM capacity for single and conjugated features using the change detection paradigm. They asked participants to memorize the colors in an array of stimuli, and manipulated the set size (i.e., the number of colored squares). After a brief interval, a second array of stimuli was presented and participants were asked to detect if the two sets of stimuli were identical. A series of experiments were conducted by repeating this sort of manipulation for objects with multiple feature conjunctions (e.g., a line segment with a particular color and orientation). It was observed that the participants were able to detect a change in sets of up to 4 objects, no matter how many features were presented in each object. They concluded that the appropriate unit of working memory capacity is the integrated object rather than the single feature. However, this object-based account of visual WM capacity has been criticized in several subsequent studies, and several factors have been found to constrain the object benefit.

### The object benefit for spatially separated features

One of the criticisms raised against Luck and Vogel’s (1997) account was that their study did not fully explain whether the benefit arises from combining multiple features into an object, or from the overlapping location of the features. Features occupied the same location in all of their experiments. For example, when colored oriented bars were used, color and orientation naturally overlapped. It is important to address the role of shared location when considering object benefit, because working memory has a limited capacity for the number of spatial locations in particular (Jonides et al., 1993; McCarthy et al., 1994), and object location can be encoded automatically regardless of task-relevancy, suggesting it has a certain primacy (Dell Acqua et al., 2010; Eimer & Kiss, 2010; Elsley & Parmentier, 2015; Kuo et al., 2009; Olson & Marshuetz, 2005). Attentional feature integration is also thought to be mediated by spatial location (e.g., Treisman & Zhang, 2006), and several studies have similarly confirmed the significant role of location in the integration of visual features in memory (Hollingworth, 2007; Saiki, 2016; Schneegans & Bays, 2017; Udale et al., 2017; Wang et al., 2016).

The question is, thus, whether the memory benefits observed by Luck and Vogel (1997) might (partially) reflect visuospatial integration. To address this, Xu (2002a, 2002b) investigated the encoding of color and orientation features of multipart objects. Xu had participants perform a change detection task in three display conditions, which relied differentially on the spatial relation between two features in an object. In one condition, two task-relevant features were located in the same part of the object. In another one, the two features were located in different parts of the same object, and in the last condition, the features were each located in different objects. The results indicated that color and orientation features that were located in the same part of the object were better encoded in visual WM, compared to features that were located in different parts of an object. It was also reported that two spatially separated features of the same object were still encoded better than two features from different objects. Despite a decline in performance when features appeared in different locations, the study demonstrated that object-based encoding benefits can be obtained, even in spatially separated parts of the same object (Xu, 2002a, 2002b).

Although Xu (2002a, 2002b) observed a memory advantage for features that were part of the same object, but which appeared in distinct spatial locations, compared to features that were part of different objects, the spatial proximity between- and within-object features was not equalized. For instance, the spatial positions of the features located in different parts of an object were much closer than the spatial positions of the features that were located in different objects. For that reason, the question is whether the benefit in the former case can truly be attributed to the object-based presentation of the features alone, or whether the recall advantage arose (also) from spatial proximity between those features. A further study of Xu (2006) addressed this issue by independently manipulating both distance and connectedness between the object parts. Memory was better for features that were closer and directly connected compared to those that were further apart and unconnected, indicating that both factors of spatial proximity and feature connectedness had a role in the object benefit in visual WM.

### Feature dimensionality and independent feature stores

Apart from the role of spatial overlap in the object benefit reported by Luck and Vogel (1997), the possible role of featural overlap has also been scrutinized. In their original study, Luck and Vogel (1997) conducted one experiment in which the object features were of the same feature dimension (color–color), and somewhat surprisingly, again found that no additional costs were involved in retaining the color conjunctions compared to maintaining the same number of objects with single colors. However, several authors failed to observe such benefits from same-dimension conjunctions and argued that features from the same dimension cannot be stored as integrated objects in visual WM (Delvenne & Bruyer, 2004; Parra et al., 2011; Wheeler & Treisman, 2002; Xu, 2002a, 2002b). Conversely, an object benefit for retaining features from different dimensions has been replicated in a considerable number of studies (Delvenne & Bruyer, 2004; Olson & Jiang, 2002; Parra et al., 2011; Riggs et al., 2011; Vogel et al., 2001; Wang et al., 2017; Xu, 2002a, 2002b).

This possible difference in memory performance with feature conjunctions within and between feature dimensions is predicted by the influential feature integration theory (Treisman & Gelade, 1980). Feature integration theory (FIT) assumes that features are first registered in memory separately from their corresponding spatial locations. In a subsequent step, the features that shared the same location then form a unitary object. Importantly, in the theory, each feature type (such as color or orientation) has its own pool of memory resources, which allows parallel processing of features, if they lie on a different dimension. Owing to this mechanism, multidimensional features can also be encoded in memory without interfering (see also Allport, 1971). By contrast, combining features of the same dimension into a single object would lead to competition for their shared memory resource, and result in a decline in the total number of features that can be recalled, contrary to the evidence originally presented by Luck and Vogel (1997). FIT furthermore predicts that attention mediates in these processes, because FIT assumes that focal attention is required to maintain integrated object representations in visual WM once the features are registered to a location. Consequently, misbinding of features across multiple objects can occur as a result of attentional distraction, or due to the absence of sustained attention (Rensink, 2000; Wheeler & Treisman, 2002; but see also Gao et al., 2010; Yin et al., 2012).

### The object benefit and the role of attention

In view of the possible role of attention, it is important to assess whether the object benefit for visual memory arises during stimulus encoding under the influence of attention, or at a later stage of information processing or maintenance in visual WM. A considerable number of studies that investigated selective attention mechanisms provide compelling evidence for the presence of object-based components of visual attention. For instance, the study of Duncan (1984) aimed to test the role of object-based attention in the perceptual processing of visual information, and in his study, two overlapping objects, a box and a line, were presented to participants. Each object consisted of two features, which varied between trials, and participants were asked to report either one or two features of the objects. The study found that reporting two features of the same object was as difficult as reporting just a single feature. However, reporting two features was harder if they belonged to separate objects, rather than to the same object. Because the spatial separation of features between and within the objects was equal, this difficulty can be interpreted as a cost of switching attention between objects. Duncan concluded that directing attention to a part of an object activated the rest of the object as well, and that all parts of the object were processed as whole.

Similarly, Egly et al. (1994) provided another demonstration of object-based attention using a cueing paradigm. They used a luminance detection task and showed two rectangular outlines to participants. In each trial, initial attention was manipulated by presenting a cue at one end of the two rectangular stimuli. In the majority of the trials the cues were valid, indicating that upcoming target squares would appear at the same end of the cued rectangle. In the rest of the trials, the cue was invalid and the target either appeared at the opposite end of the cued rectangle, or at the equivalent distance end of the uncued rectangle. Invalid cues required the participants to relocate their attention from the cue location to the target location, and the study focused on the response latency in invalid cue trials, reflecting the time cost of attention shifts within and between objects. They observed that the cost of switching attention between objects was larger than the cost of shifting attention within the object, demonstrating an object-specific benefit of the attention. Overall, these studies suggest that attention can select visual information based on objects. It is natural to assume that object-based information can consequently be better encoded and recalled.

The functional relationship between object-based attention and memory has also been exemplified in interference tasks. For instance, Matsukura and Vecera (2009) gave participants an attention task to perform, while they were concurrently retaining either an object or location memory. The results indicated that concurrent object-based attention tasks interfered more with object memory than with spatial memory, and this finding was interpreted to mean that some forms of object-based selection and object-based memory might be processed by the same mechanism. Similarly, another study by Barnes et al. (2001), using a dual-task paradigm found that object-based advantages for selective attention decreased while maintaining an object memory, but also that no interference occurred while maintaining a verbal or spatial memory.

Another significant role of attention for WM is that attention can prioritize items during encoding, while items in memory may also bias attention. It has been assumed that attention assists information entry into visual working memory (Bays & Husain, 2008; Schmidt et al., 2002), and can, thus, increase the probability of information being maintained in visual working memory for further processing. This attentional prioritization of WM items was observed not only when directing attention to the target item before it appeared (pre-cueing), but also afterwards (retro-cueing), and during the appearance of stimuli (Griffin & Nobre, 2003; Landman et al., 2003; Matsukura et al., 2007; Pertzov et al., 2013; Schmidt et al., 2002).

Furthermore, memory maintenance may be enhanced by retro-cueing, which may protect a memory representation from decay or interference (Makovski et al., 2008; Souza et al., 2016). Pre-cueing similarly not only prioritizes encoding the target item into WM, but also facilitates the maintenance of its memory representation (Ravizza et al., 2016; Schmidt et al., 2002). Therefore, considering the relationship between attention and visual WM, as well as the object-based theory of attention, in which attention directed to a part of an object automatically extends to the whole object, it is natural to conclude that object-based information can be both better encoded and recalled when a part of the object is prioritized.

### The present study

In the present study, we aimed to further elucidate the nature of the object benefit in visual WM. We examined whether multiple pieces of visual information (i.e., visual features) that appear within the same object are more efficiently maintained in WM than those that are part of separate objects. In the first experiment, we assessed the strength of the object effect for pairs of orientation features presented at equidistant locations within and across simple objects made up out of first order contours. In the second experiment, we subsequently examined the object benefit for pairs of features from different dimensions (color and orientation). In the third experiment, we tested potential object benefits that may occur due to changes in the location of the object and its features in each trial, when the object was no longer the only spatial reference point in the display. Finally, in the fourth experiment, we examined whether the object benefit is affected by strategic use of information that is related to the object. Our expectations for the collective experiments were that an object benefit should exist even for equally spaced features, that the object benefit should be larger for non-interfering features, that is, those from different dimensions, that the size of the object effect may diminish by reducing attention to the objects themselves (i.e., the contours), and that strategic usage of information during visual processing may contribute to the effect, but not fully account for it.

## Experiment 1

This experiment aimed to determine whether the presentation of orientation features within simple contour shapes benefits the encoding, maintenance, or recall of representations in WM. To achieve this goal, we used three oriented grating stimuli, presented at equidistant locations. Two of these were displayed as part of an object by embedding them together in a gray ellipse shape, while the third one was embedded in a gray circular shape on its own. One of the stimuli in the large object was colored red, indicating this stimulus would always be the first target. The second target was one of the two remaining stimuli. Although participants knew the first target stimulus during visual processing, they did not know which of the remaining stimuli would be the second target and thus, they had to memorize all three stimuli to be successful in both responses. This created two experimental conditions, depending on whether the second target stimulus was inside the same object with the first target stimulus, or outside, in the separate object. We expected better memory performance on the second target when it was in the same object as the prioritized (first) item. This hypothesis was motivated by the fact that attention prioritizes objects and this benefit would improve the quality of the encoding for features belonging to the same object. Likewise, attending to the first target, which was always in the large object, could induce memory benefits to features within the same object via the spread of attention.

### Method

#### Participants

Twenty-five first year psychology students (21 females, mean age = 19.2, range = 18–20) with normal or corrected-to-normal vision were recruited from the University of Groningen. Prior to the experiment, participants signed an informed consent form, and they were naive to the purpose of the study. All students were rewarded course credits for their participation. The study was approved by the Ethical Committee Psychology (approval number 1920-S-0071) and it was conducted in accordance with the Declaration of Helsinki (2008).

#### Apparatus and stimuli

The experiment was programmed in Matlab (version 2017, 64bit), using the Psychtoolbox extension (Brainard, 1997; Kleiner et al., 2007; Pelli, 1997). Stimuli were displayed on a 27" LCD monitor using a standard desktop computer. The screen was set at a refresh rate of 100 Hz and a resolution of 1920 by 1080 pixels in 16-bit color. The participants were tested in an experimental room with uncontrolled but normal interior lighting, and they were seated at about 60 cm viewing distance from the monitor. All behavioral responses were collected via a standard USB keyboard.

The stimuli were three sine wave gratings (radius 2.1° of visual angle, 1 cycle/° and 50% contrast). The center points of each grating were placed equidistantly from each other on the circumference of an invisible circle (3.74° radius) to form the corner points of an invisible equilateral triangle in the center of the screen. In each trial, the invisible triangle was rotated over the invisible circle between 1 and 360 degrees in steps of 1 degree, and presented in random order without repetition. One of the gratings was presented in red (RGB = [200 128 128], luminance 185 cd/m^2^) to point out the first target grating. The other two gratings were monochrome (RGB = [128 128 128], luminance 160 cd/m^2^). The red grating and one randomly selected monochrome grating were enclosed by an oval shape whose width and height were 14.49° and 4.83°, respectively. The third, remaining grating was enclosed by a circular shape with a diameter of 4.15°. Both shapes were gray (RGB = [100 100 100], luminance 127 cd/m^2^), and outlined by a 0.09° wide black contour. This ensured that any differences in memory performance cannot be attributed to contrast or luminance. In addition to the stimuli presented at the memory display, two gray circles with a black outline were later presented at corresponding target locations as feedback circles. They were the same size as the targets and rendered in the same gray as the shapes.

The orientation of each grating was independently chosen at random from the range of angles 0°–180° without repetition between trials, in steps of 0.56° (180°/total trials). Throughout the entire trial, a black dot with a white edge (radius 0.83°) was presented in the center of the screen as a fixation point, and participants were instructed to maintain their gaze on the fixation dot. All the stimuli were displayed against a uniform light gray background (RGB = [128 128 128], luminance 160 cd/m^2^), which was maintained during the entire experiment. Note that all luminance and color values are approximations of the Psychtoolbox texture function and Memtoolbox color wheel RGB values.

#### Procedure

**Fig. 1:**
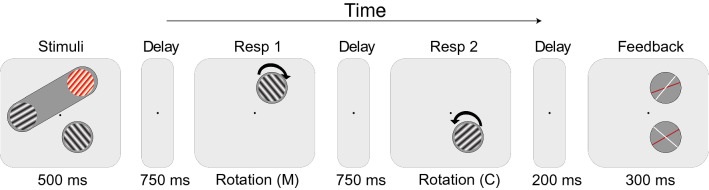
An example of the single-trial sequence used in Experiment 1. Three oriented gratings were presented to participants to memorize. Subsequently, two of them were sequentially probed. The red grating and one of the gray gratings were always enclosed by an oval shaped object, and the third one was enclosed in a circular shaped object. The orientation of the red-colored grating was always tested first and one of the other gratings was tested second. After the second response, a feedback screen consisting of two lines for each stimulus was shown. White lines depicted the actual orientation of the gratings and red lines depicted the participant’s response

The sequence of a trial is shown in Fig. 1. Each trial started with the presentation of a fixation dot in the center of the screen for a duration of 700 ms. Then, three grating orientations were presented in the memory display for a duration of 500 ms. The red grating was always presented inside the flat oval object shape, together with one of the two other gratings. The task required the participant to memorize all orientations for the following memory recall test. The participants were instructed that the orientation of the red grating would always be tested first, and that one of the monochrome grating’s orientations would be tested second. After presentation of the gratings, there was a 750-ms delay period and then the first response probe with a random orientation was presented at the location of the corresponding target stimulus. To match the orientation of the probe to the memorized orientation, participants could use the keyboard; pressing the ‘C’ key for clockwise rotation, the ‘*M*’ key for counterclockwise rotation, and pressing the space key to submit the response. The response probe stayed on the screen until a response was given. Once the first response was submitted, there was another 750 ms delay period. Following the delay, the second response probe for one of the other gratings was presented at the location of the corresponding target stimulus. Participants were, thus, asked to reproduce the orientation of the grating that had been presented on the location of this second probe in the memory display.

Trials were equally divided between the two object conditions. In half of the trials the second target was inside of the object containing the first target (T2 in), in the other half second target was outside of the object containing the first target (T2 out). The trial types were presented in a randomized order. Following a brief 200 ms interval, a feedback screen was shown for 300 ms. The feedback screen consisted of two lines for each grating stimulus, represented inside circles at their corresponding locations. One of these lines was white, representing the actual orientation, and the other line was red, showing the participant's response. The distance between these lines, thus, indicated exactly how much the response deviated from the correct orientation of each grating.

The experiment was made up of 320 trials, divided into 20 blocks of 16 trials. At the end of every block, participants were able to see their average accuracy (based on the number of responses within or outside of 20° away from the real target value) for the first and second targets separately (the latter independent of its location), for the blocks that had been completed so far. They were shown performance for each block as well as the overall average of all completed blocks. Participants were given the opportunity to take a short break between the blocks. Prior to the experimental trials, all participants performed 4 blocks of practice trials, with 8 trials per block for a total of 32 trials, to become familiar with the task.

#### Data analysis

Two sets of analyses were performed. First, we calculated the average response accuracy for each participant. Responses that had an absolute deviation of 20° or less compared to the target were defined as correct responses. This criterion served as a basis for the preliminary analyses to see whether there were overall differences between object conditions, and was also used to give participants feedback on their performance at the end of each block. Then, we used Bayesian paired sample t-tests to compare the average accuracy for the two object conditions, which corresponded to having the second target inside versus outside the object containing the first target. These tests were conducted for the first and second response separately. These analyses were performed in JASP using version 0.16.3 (JASP Team, 2022). The approach of Wetzels et al. (2011) was used for interpretation of Bayes Factors (BF). According to this interpretation, BF_10_ values between 1 and 3 were classified as anecdotal evidence, 3 and 10 as substantial evidence, 10 and 30 as strong evidence, 30 and 100 as very strong evidence, and BF_10_ values above 100 as decisive evidence in favor of the alternative hypothesis. BF_10_ values between 0.33 and 1 were classified as anecdotal evidence, 0.1 and 0.33 as substantial evidence, 0.03 and 0.33 as strong evidence, 0.01 and 0.03 as very strong evidence, and BF_10_ values below 0.01 as decisive evidence in favor of the null hypothesis.

The second analysis was based on the Standard Mixture model, which is described by Zhang and Luck (2008). The Standard Mixture model assumes that there are two possible types of responses that can be given by participants. These are either responses given based on the memory of the target (*P*^M^) or pure guessing (1 − *P*^M^). When participants do have a memory of the target, their responses should have errors of variability around the target value, which is called memory precision (*σ*). Before performing the analysis, we computed angular deviations between participants’ response and the true orientation of targets, which fall between ± 90°, and where 0° represents the true orientation of the target. To implement the Standard Mixture model, we used the CatContModel package (Version 0.7.0; Hardman et al., 2017). This model has a hierarchical nature and individual participants are seen as samples of the population. Two steps were taken to implement the model-based analysis. First, multiple models were compared to determine the best fitting model. We used both the full and the reduced models; these models differ on which model parameters are constant across conditions. In the full model, both *P*^M^ and *σ* were allowed to vary between object conditions. Only one of the parameters was kept constant across object conditions in the second and third models (*P*^M^ and *σ*, respectively). Both parameters were kept constant in the last model. The model fits of CatContModel variants were compared using the Watanabe–Akaike Information Criterion (WAIC), which is considered to be the most appropriate fit statistic for hierarchical Bayesian models (Hardman et al., 2017). WAIC is based on the overall likelihood of the model and has a penalty term for the effective number of free parameters (Gelman et al., 2014). Smaller WAIC scores indicate better model fit. All parameter values were estimated by Bayesian Markov Chain Monte Carlo (MCMC) approaches. The data of all conditions and participants were fitted simultaneously and 11,000 iterations (with 1000 burn-in) were run for each model. After the first step of selecting the best model, we performed the hypothesis tests for comparing parameters that were estimated by the best model. The model statistic is based on Bayesian tests that are conceptually equivalent to ANOVAs (see Ricker & Hardman, 2017). We obtained subject-level parameter estimates from posterior chains of the best model parameters. For interpretation of the Bayes Factors (BF), we again followed the approach of Wetzels et al. (2011). The “ggplot2” package (Wickham, 2016) was used for the visualization of analysis results.

### Results

**Fig. 2:**
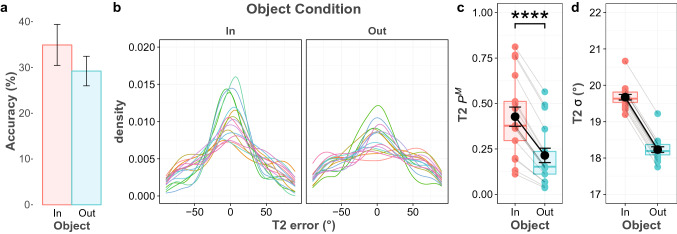
T2 performance in Experiment 1. **a** The bar graph shows T2 accuracy for two object conditions. Error bars depict 95% confidence intervals. **b** Probability density plot of the T2 error distribution, shown separately for each object condition and participant, which are indicated by different colors. **c** Probability of having T2 in memory (P.^M^) based on the best model predictions for all participants and object conditions. Each of the colored circles represents the mean for an individual participant and gray lines connect each participant’s mean across the object conditions. The black circle represents the mean across participants and error bars show the standard error of the mean**.** The thick horizontal lines in the colored boxes represent median and quartile values. **d** Precision of the T2 memory representation (*σ*) based on the best model predictions. Asterisks denote evidence against the null hypothesis (* moderate evidence, ** strong evidence, *** very strong evidence, and **** decisive evidence)

First, we analyzed the average accuracy of both targets. Overall accuracy for the first target was 84.2%, while that of the second target was much lower at 32.1%. A paired sample t-test was conducted to compare the average accuracy for the two object conditions. For the first target, performance accuracy averaged 84.5% in the T2-in condition, and 84% in the T2-out condition. The statistical test revealed substantial evidence in favor of the null hypothesis (BF_10_ = 0.275), indicating that the first response was equally accurate in both conditions. For the second response, the average accuracy was 34.9% in the T2-in condition and dropped to 29.2% in the T2-out condition (as shown in Fig. 2a). Test results revealed decisive evidence in favor of the alternative hypothesis for the effect of Object (BF_10_ > 100).

Second, we performed mixture model analysis, and the Constant *σ* and *P*^M^ across Object model was selected as the best model, since it had the lowest WAIC value in the model comparisons for the first response. Table 1 shows all model fits, in which a smaller WAIC indicates a better fit. This indicated that varying either the probability of recall or the memory precision parameter did not improve the model. For the second response, the Full model was the best model and it indicated that both probability of recall and memory precision varied across object conditions. Figure 2c and d show both parameter plots for the second response. Further, Bayesian analysis provided that there was decisive evidence that recall probability was higher when the second target was inside the same object with the first target (BF_10_ > 150). This result indicates that presenting two targets into the same object led to an increase in probability that a second target was present in working memory; however, it did not reliably affect the precision of the memory representation (BF_10_ = 0.737).

**Table 1:** WAIC for all tested models in experiment 1

Model	T1		T2	
	WAIC	Difference from the best model	WAIC	Difference from the best model
Full model	12,102.985	3.49	**28,440.68**	**0.00**
Constant * σ* across object	12,102.09	2.60	28,441.14	0.46
Constant * P*^M^ across object	12,101.155	1.67	28,468.22	27.54
Constant * σ* and * P*^M^ across object	**12,099.49**	**0.00**	28,507.22	66.54

In the full model, both *P*^M^ and *σ*^o^ parameters vary across object conditions, in the constant *σ* and constant *P*^M^, one parameter (*P*^M^ or *σ*^o^) was constant, while the other parameter varied across object conditions, and only the varying parameter was freely estimated for each object condition. In the Constant *σ* and *P*^M^ model, both parameters were constant across object conditions and a single *P*^M^ and *σ* were estimated across object conditions. Lower values indicate better fit and the best model is printed in bold

## Experiment 2

In Experiment1, VWM was examined for objects defined by a single feature dimension (orientation), to test whether these features can be more efficiently maintained as a result of the information being bound in a single object. In Experiment 2 we implemented the same experimental procedure for objects containing features from two different dimensions (color and orientation), to investigate whether feature conjunctions from different feature dimensions can further enhance memory performance, due to the use of independent memory stores for each feature dimension, as is proposed in FIT (Treisman & Gelade, 1980).

### Method

#### Participants

Twenty-eight new psychology students (nineteen females) participated in the experiment (mean: 21.6 years, range 19–32).

#### Apparatus, stimuli, design, and procedure

**Fig. 3:**
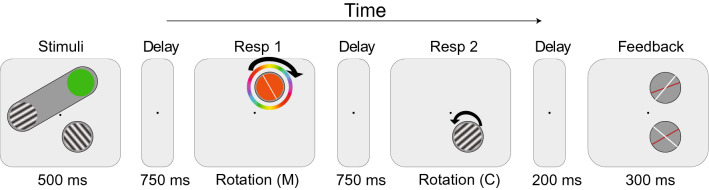
An example of the single-trial sequence used in Experiment 2. The red-colored grating was changed to a colored circle and participants were instructed to memorize one color and two orientation features. The color was always tested first, and one of the orientations second

The experimental setup was identical to Experiment 1, except for the changes that are detailed below. The first tested stimulus was changed from an orientation grating to a colored circle, which had to be reproduced, as shown in Fig. 3. The other stimuli were the same as in previous experiments. In each trial, the first target color was randomly selected from 180 possible RGB values drawn from the color wheel of Memtoolbox (Suchow et al., 2013). As indicated, these displayed colors were an approximation of the true color wheel.

In the first response display, a color circle (4.5° radius and 0.35° thick outline) was presented around the probe location, with its fill displaying a random color at onset. All colors were evenly distributed along one half of the color wheel, the other half was its flipped mirror image, so that both ends of the probe line for the color response pointed to the same color and had the same angular orientation range as on the orientation probe. The initial color of the probe was randomly chosen on each trial and participants were asked to rotate the probe to the desired color on the color wheel. The orientation of the color wheel was randomized each trial. The total number of trials was increased to 360 to present all possible stimulus locations, and to sample all orientations/colors evenly.

### Results

**Fig. 4:**
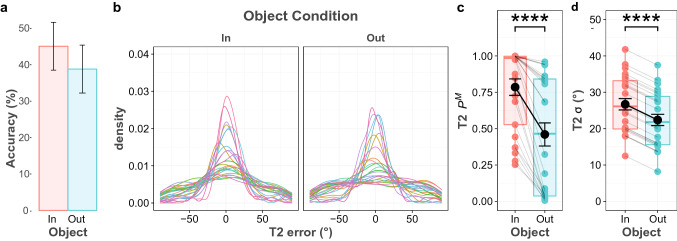
T2 performance in Experiment 2. **a** T2 accuracy for both object conditions **b** Probability density plot of the T2 error distribution for all participants and object conditions. **c** Probability of having T2 in memory (*P*^M^), **d** Precision of the T2 memory representation (*σ*), based on the best model prediction

The average accuracy for the first target was 92.4%, and similar to the results of Experiment 1, the average accuracy for the second target (41.9%) was considerably lower. The Bayesian paired sample *t*-tests revealed that there was anecdotal evidence that the two object conditions did not differ for the first response (BF_10_ = 0.451), and average accuracy was 92% in T2-in and 92.8% in T2-out conditions. For the second response, accuracy averaged 45.1% in the T2-in condition and decreased to 38.8% in the T2-out condition (Fig. 4a). Test showed that there was anecdotal evidence for a difference between the two object conditions (BF_10_ = 1.451).

For the mixture model analyses, the data of the first response fitted best in the Constant *σ* and *P*^M^ across Object model (Table 2). On the other hand, the Full Model was the best model to fit the data of the second response, which contains effects of the object both on probability of recall and on memory precision. Bayesian analysis provided extreme evidence for an object effect; when the second target was inside the object together with the first target, its recall probability increased (BF_10_ > 100), while its memory precision decreased BF_10_ > 100). This result implies that object-based presentation of two targets increases the probability of the second target being maintained in working memory (Fig. 4c). However, the memory precision of the second target decreased at the same time (Fig. 4d).

**Table 2:** WAIC for all tested models in experiment 2

Model	T1		T2	
	WAIC	Difference from the best model	WAIC	Difference from the best model
Full model	9523.942	2.06	**33,117.2**	**0.00**
Constant * σ* across object	9522.272	0.39	33,180.09	62.89
Constant * P*^M^ across object	9523.04	1.16	33,288.73	171.53
Constant * σ* and * P*^M^ across object	**9521.883**	**0.00**	33,297.79	180.59

We also compared results from Experiment 1 with Experiment 2 using Bayesian mixed factorial ANOVA, to determine whether the object benefit differed between objects containing a single feature dimension, and those containing features of different dimensions. For this comparison, we looked at the probability of having T2 in memory because a significant object effect was observed for the probability of recall in both experiments. Therefore, the experiments were treated as between-subject factor, object condition was treated as within-subject factor, and probability of recall was treated as dependent variable. There was decisive evidence in favor of the alternative hypothesis that main effects existed for both Experiment (BF_inc-10_ > 100) and Object (BF_inc-10_ > 100), and strong evidence for their interaction (BF_inc-10_ = 17.241). This interaction suggested that the probability of having T2 in memory for an object defined with features from different dimensions (Experiment 2) was larger than for an object defined with single dimensional features (Experiment 1).

## Experiment 3

In the previous two experiments, the locations of the objects and the features were changed on each trial, rotating randomly over the invisible circle. This variation in location might modulate how the stimuli were perceived and organized in VWM, as in this design, the object is the only spatial reference point in the display. This may also draw more attention to the objects surrounding the stimuli than might be the case in situations in which other spatial references are available. In other words, the location-variable design might increase the possibility of the object and feature parts being perceived as a unified whole, thereby enhancing the object benefit. To assess the magnitude of this potential effect in Experiment 3, we presented all three features in fixed locations, and only the object surrounding those features was changed between trials. We expected participants to focus more on the locations of the features themselves, which might reduce the object effect, or even make it disappear completely.

### Method

#### Participants

Nineteen students (15 females; *M* = 23.1 years old) took part in this experiment for course credit or 10 euro.

#### Apparatus, stimuli, design, and procedure

The procedure for the experiment was the same as that of Experiment 1, except that the grating stimuli and the first target were shown at three fixed locations. At the beginning of the experiment, three equidistant locations were randomly selected from 360 possible locations on the circumference of an invisible circle as the center point of the gratings, like in previous experiments, however, they were presented in these locations throughout the experiment, instead of being rotated over the invisible circle on each trial. To eliminate advantages of visual processing for a stimulus presented in one location over another one, the first target was shown in one of these three locations for the first one third of the trials, then it switched to another fixed location after the next one third of the trials, and again for the last one third of trials. Since the targets were, thus, shown evenly across all three locations, any potential benefits related to specific locations should be canceled out.

### Results

**Fig. 5:**
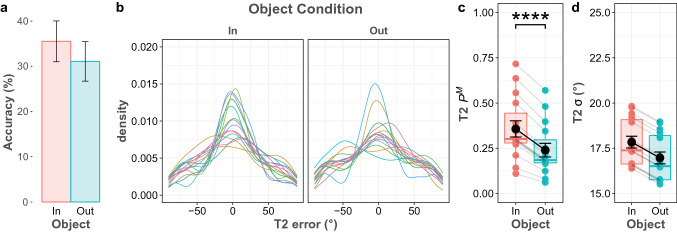
T2 performance in Experiment 2. **a** T2 accuracy for both object conditions **b** Probability density plot of the T2 error distribution for all participants and object conditions. **c** Probability of having T2 in memory (*P*^M^), **d** Precision of the T2 memory representation (*σ*), based on the best model prediction

There seemed to be some decline in the accuracy of the first target (79.8%) compared to that in the previous two experiments, while the accuracy of the second target (33.9%) remained similar to the first experiment. There was substantial evidence that the response accuracy of the first target did not differ between the two object conditions (BF_10_ = 0.246). The accuracy of the second response was again higher when the first and second targets were part of the same object, Bayesian test revealed substantial evidence in favor of effect of object (BF_10_ = 7.173). Average accuracy of the second response was 35.5% when the second target was inside the same object with the first target, and 31.1% when it was presented in a separate object (Fig. 5a). We also tested for potential effects of switching the target position, and found no significant switching costs (see the supplementary materials for detail of this analysis).

Analysis of the mixture model for the first response again showed that both recall probability and memory precision were constant between the object conditions (Table 3). Further, the second responses were best fit to the Constant *σ* across Object model, in which probability of recall varies across object conditions, while memory precision was constant. According to Bayesian analysis, there was very strong evidence that recall probability of the second target was higher when both targets had been presented in the same object (BF_10_ > 100).

**Table 3:** WAIC for All tested models in experiment 3

Model	T1		T2	
	WAIC	Difference from the best model	WAIC	Difference from the best model
Full model	13,287.472	4.52	25,540.19	0.25
Constant * σ* across object	13,286.103	3.15	**25,539.94**	**0.00**
Constant * P*^M^ across object	13,284.805	1.85	25,558.36	18.42
Constant * σ* and * P*^M^ across object	**13,282.955**	**0.00**	25,560.61	20.67

To compare the magnitude of the object effect on the probability of having T2 in memory between Experiment 1 and Experiment 3, we performed Bayesian mixed factorial ANOVA. This analysis revealed decisive evidence for the main effect of Object (BF_inc-10_ > 100), and no evidence was found for the main effect of Experiment. There was also anecdotal evidence in favor of the alternative hypothesis that Object and Experiment interacted (BF_inc-10_ = 1.957). According to this interaction result, it appears that giving features in fixed location decreased the object effect of recall probability in Experiment 3. However, the object benefit did not disappear completely.

## Experiment 4A

So far, the results from all three experiments confirmed that being embedded in the same object enhances the chance of recalling features from equally spaced stimuli that were made up from a single dimension, or from two dimensions (color and orientation), both when their locations were rotated or kept constant. However, the experiments did not indicate to which extent this object benefit arose from strategic choices, aimed at using object-related information to facilitate feature processing. Experiment 4A was designed to address this question. We conducted an experiment using three different presentation orders: Objects either appeared before or after the grating stimuli, or were presented simultaneously as in the previous experiments. If the object benefit is (partially) due to strategic choices, then participants should be able to use object information to guide processing, even if it is temporally displaced; either as a kind of pre-cue (e.g., to direct attention), or even as post-cue (e.g., to structure information in VWM).

### Method

#### Participants

Twenty-three students (12 females; *M* = 22.43 years old) participated in the experiment. Participation was compensated with course credit or money (14 euro).

#### Apparatus, stimuli, design, and procedure

**Fig. 6:**
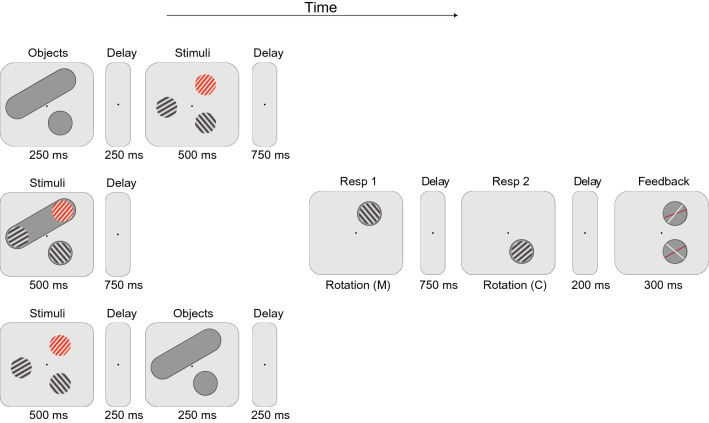
The three trial types used for Experiment 4A and 4B. The top trial flow shows the Before condition, where objects were presented before the grating stimuli, the middle trial flow shows the Simultaneous condition, where object and grating stimuli were presented simultaneously as in previous experiments, and the bottom trial flow shows the After condition, where objects were presented after the grating stimuli

The setup of the experiment was the same as Experiment 1, except for the following changes. In addition to the two object conditions (in/out), as implemented in the previous experiments, three different presentation orders were created (Fig. 6): The object shape could appear before, after, or simultaneous with the gratings; with the latter condition replicating the design of the previous experiments. Objects that were presented separately from the grating stimuli, both in the Before and After trials, lasted 250 ms and were followed by a 250-ms period, which was inserted between the objects and the grating stimuli. In the Before and Simultaneous trials, the grating stimuli were followed by a 750-ms delay, after which the first probe was presented. In the After trials, the grating stimuli were followed by a 250-ms interval, after which the objects were presented for 250 ms. Another 250-ms delay was then given before the first probe to equalize the period between the onset of the stimuli and probe presentation for all three conditions.

The total number of the trials was increased to 540, and they were equally divided among the three object order conditions; 180 trials each. Again trials in each object condition were equally divided between conditions also, resulting in 90 trials per each possible combination of two within-subject conditions. All trial types were presented in a random order. To prevent participants getting fatigued by the increased trial count, the experiment was completed in two sessions. Each session comprised 270 trials split into 9 blocks. Participants could complete both sessions on the same day or on two consecutive days. When both sessions were completed on the same day, they were separated by a compulsory break of at least one hour.

For the analysis of accuracy, we used a Bayesian Repeated-Measures ANOVA to test the main effect of Object, Order, and their interaction on the first and second target. These analyses were performed in JASP with default settings. Inclusion Bayes factor (BF_inc_) based on matched models is reported for ANOVA main effects and interactions. Inclusion Bayes factors provide a measure of how well the data supports the inclusion of a factor in the model by comparing models with a particular predictor with models that exclude that predictor. Post hoc tests were corrected by fixing prior probability to 0.5 (Westfall, Johnson, & Utts, 1997). For model analysis, the data of all conditions and participants were fitted simultaneously using a factorial design with the factors Order (Before, Simultaneous, and After) and Object (In or Out). We compared the model with both main effects (Object and Order) and their interaction. Three parallel chains of 11,000 iterations (1000 burn-in) were run for each model and then their posterior distributions were combined.

### Results

Overall average accuracy was 85.8% for the first response and 40.1% for the second response. Bayesian repeated-measures ANOVA results showed decisive evidence in favor of the alternative hypothesis that there was a main effect of Order (BF_inc-10_ > 100), there was substantial evidence against the main effect of object (BF_inc-10_ = 0.231) and its interaction with object order (BF_inc-10_ = 0.124). Post hoc analysis showed there was decisive evidence that the accuracy in the Before condition was higher than in the Simultaneous (BF_10_ > 100) and the After conditions (BF_10_ > 100). In the Before condition, accuracy averaged 88.7%, compared to 84.4% in the Simultaneous condition, and 84.6% in the After condition.

**Fig. 7:**
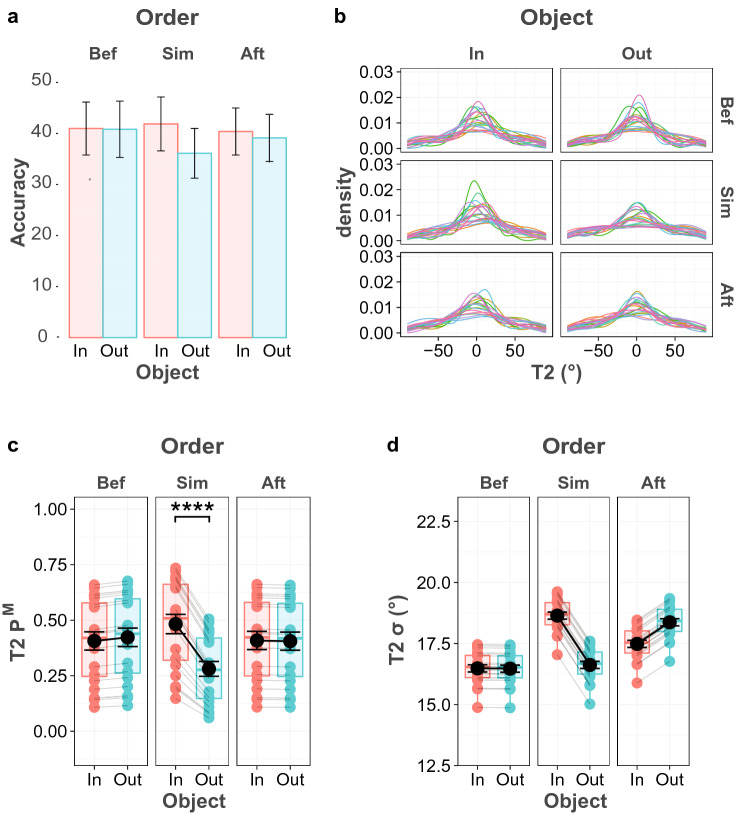
T2 performance in Experiment 4A. **a T2** accuracy for each object and order condition **b** Probability density plot of the T2 error distribution for all participants and conditions. **c** Probability of having T2 in memory (*P*^M^), **d** Precision of the T2 memory representation (*σ*), based on the best model prediction

For the second response, the statistical test revealed anecdotal evidence for main effect of Object (BF_inc-10_ = 1.019), no evidence for main effect of Order, and very strong evidence for their interaction (BF_inc-10_ = 66.844). Figure 7a plots the mean accuracy of the second response as a function of order and object condition. Post hoc comparison revealed strong evidence for an object effect under the condition where the object and the grating stimuli were presented simultaneously (BF _10_ = 17.700), but no difference was found for either the Before (BF _10_ = 0.355) or After condition (BF _10_ = 0.284).

All the model fits for both responses are listed in Table 4. For the first response, recall probability and memory precision only varied across presentation order but not across object conditions. There was strong evidence for a main effect of Order on memory precision (BF_10_ = 11.45), and anecdotal evidence for an effect on recall probability (BF_10_ = 2.56). No evidence was found for an effect on memory precision of both the Object condition and its interaction with Order. The pairwise comparison of experimental conditions provided decisive evidence that presenting objects before the memory stimuli increased memory precision compared to when the objects were presented simultaneously (BF_10_ > 100) and after these stimuli (BF_10_ > 100). There was strong evidence that recall probability of the first target was higher in the Before condition compared to the After condition (BF_10_ = 41.6) and moderate evidence compared to the Simultaneous condition (BF_10_ = 6.8).

**Table 4:** WAIC for all tested models in experiment 4A

Model	T1		T2	
	WAIC	Difference from the best model	WAIC	Difference from the best model
Full model	18,782.78	11.52	42,598.69	2.92
Constant * σ* across object	18,799.13	27.86	42,598.24	2.48
Constant * σ* across order	18,800.69	29.43	**42,595.7**	**0.00**
Constant * P*^M^ across object	18,776.44	5.17	42,619.96	24.19
Constant * P*^M^ across order	18,776.03	4.77	42,615.19	19.43
Constant * σ* across object and …				
Constant * σ* across order	18,791.4	20.14	42,596.74	0.97
Constant * P*^M^ across object	**18,771.26**	**0.00**	42,630.74	34.98
Constant * P*^M^ across order	18,786.16	14.89	42,618.2	22.44
Constant * P*^M^ across object and order	18,785.14	13.88	42,629.39	33.63
Constant * P*^M^ across object and …				
Constant * σ* across order	18,796	24.74	42,627.01	31.25
Constant * σ* across object and order	18,794.28	23.01	42,629.74	33.98
Constant * P*^M^ across order	18,789.28	18.01	42,615.36	19.59
Constant * σ* across order…				
Constant * P*^M^ across order	18,824.8	53.54	42,626.02	30.25
Constant * P*^M^ across object and order	18,822.49	51.23	42,635.77	40.01
Constant * P*^M^ across order…				
Constant * σ* across object and order	18,823.72	52.45	42,625.84	30.07
Constant * σ* and* P*^M^ across object and order	18,820.87	49.60	42,637.35	41.58

For the second response, the Constant *σ* across Order model that only contains effects of order and object condition on recall probability was selected as the best fitting model, as indexed by WAIC (Table 4). Figure 7c and d show the parameter plots of this model. In line with the model selection, only the main effects and interactions on memory probability (*P*^M^*)* were tested, and the tests revealed decisive evidence for a main effect of Object (BF_10_ > 100), and strong evidence for an interaction effect with Order (BF_10_ = 11.52), while no main effect of Order was found (BF_10_ = 0.11). Furthermore, pairwise comparisons showed that there was extreme evidence for an effect of Object when the object and memory stimuli were presented simultaneously, (BF_10_ > 100), which indicated that recall probability was higher for the within-object condition than for the outside-object condition. However, no difference was found between the object conditions for the other two orders.

## Experiment 4B

In Experiment 4A, an object benefit was found only for the simultaneous display condition, and not for the other two temporally separated display conditions. This might indicate that the participants were unable to make strategic use of the appearance of the object. However, in Experiment 4A the presentation order varied randomly from trial to trial. This might have made it too difficult for the participants to adapt their processing mode and to make effective use of the object ‘pre’- and ‘retro’-cues. To eliminate this potential difficulty, in Experiment 4B, presentation order was kept constant within trial blocks, which should maximize the opportunity to adapt strategically to the different presentation orders.

### Method

#### Participants

Twenty-nine students (*M* = 21.7 years old; 19 females) took part in this experiment in exchange for course credit or 14 euros.

#### Apparatus, stimuli, design, and procedure

The procedure was identical to that of Experiment 4A, with the exception that the trial types for presentation order were set as a block design. Each presentation order was used in six consecutive blocks. The order of the six-block triplets was counterbalanced across participants.

### Results

**Fig. 8:**
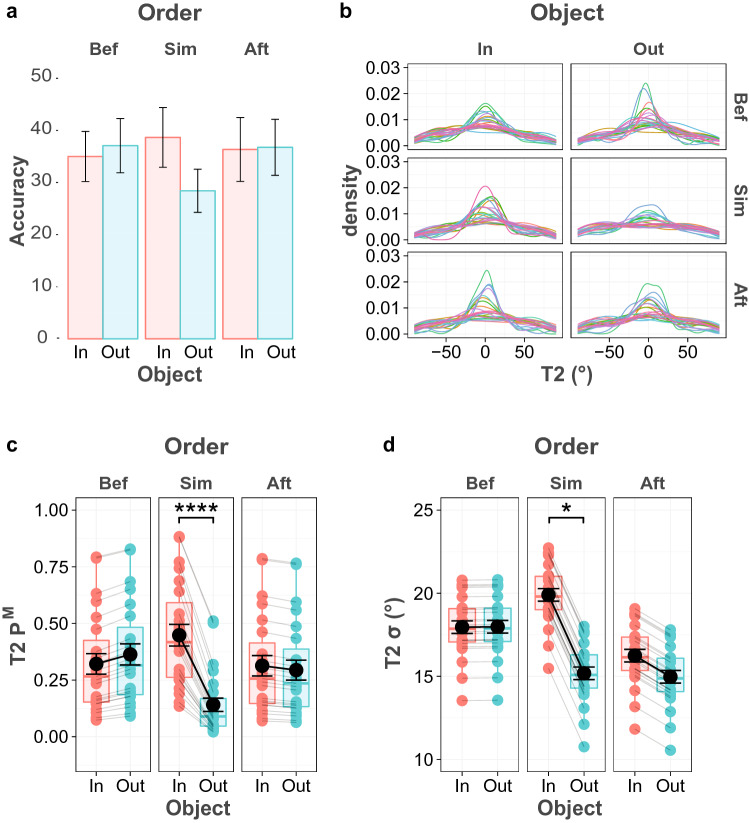
T2 performance in Experiment 4B. **a** T2 accuracy for each object and order condition **b** Probability density plot of the T2 error distribution for all participants and conditions. **c** Probability of having T2 in memory (*P*^M^), **d** Precision of the T2 memory representation (*σ*), based on the best model prediction

The average first response accuracy was 83%. There was substantial evidence observed in favor of the null hypothesis for the main effect of Object (BF_inc-10_ = 0.208) and an interaction effect of Order and Object (BF_inc-10_ = 0.145), and no evidence observed for main effect of Order. The overall mean score of the second response was 35%, T2 mean accuracy in all conditions is plotted in Fig. 8a. There was no evidence for main effect of Order and main effect of condition, however, there was decisive evidence for their interaction (BF_inc-10_ > 100. Post hoc tests revealed that there was decisive evidence that the average accuracy in the Simultaneous condition was significantly higher for within-object than for outside-object trials (BF_10_ > 100).

Next, the comparison of mixture models indicated that the best fitting model for the first target was Constant *P*^M^ and *σ* across Object (Table 5). As in the previous experiment, memory probability and memory precision only varied across presentation order but not object conditions. The statistical test produced anecdotal evidence for a main effect of Order on recall probability (BF_10_ = 2.64), and on memory precision (BF_10_ = 1.4). Pairwise comparisons yielded extreme evidence that the recall probability of the first target increased when the object was presented before the memory stimuli, compared to when it was presented after (BF_10_ > 100). There was also very strong evidence that memory precision of the first target in the Before condition was higher than in the Simultaneous condition (BF_10_ = 50.1).

**Table 5:** WAIC for all tested models in experiment 4B

Model	T1		T2	
	WAIC	Difference from the best model	WAIC	Difference from the best model
Full model	25,614.39	6.36	**54,976.5**	**0.00**
Constant * σ* across object	25,625.68	17.66	54,985.8	9.26
Constant * σ* across order	25,612.19	4.17	54,982.99	6.45
Constant * P*^M^ across object	25,610.98	2.96	55,032.69	56.16
Constant * P*^M^ across order	25,631.44	23.42	55,034.32	57.78
Constant * σ* across object and …				
Constant * σ* across order	25,624.43	16.41	54,991.09	14.55
Constant * P*^M^ across object	**25,608.02**	**0.00**	55,085.06	108.52
Constant * P*^M^ across order	25,628.8	20.77	55,071.71	95.17
Constant * P*^M^ across object and order	25,627.26	19.23	55,092.68	116.14
Constant * P*^M^ across object and …				
Constant * σ* across order	25,622.33	14.30	55,088.84	112.31
Constant * σ* across object and order	25,620.24	12.22	55,088.53	111.99
Constant * P*^M^ across order	25,629.89	21.87	55,038.56	62.02
Constant * σ* across order…				
Constant * P*^M^ across order	25,650.82	42.79	55,073.99	97.45
Constant * P*^M^ across object and order	25,647.57	39.55	55,107.16	130.63
Constant * P*^M^ across order…				
Constant * σ* across object and order	25,648.11	40.09	55,080.15	103.61
Constant * σ* and * P*^M^ across object and order	25,645.64	37.62	55,105.58	129.04

The best fitting model for the second target included both an effect of Object and of Order on both the probability of recall and memory precision (Full Model). Both parameters are plotted in Fig. 8c and d. For the probability to recall the target item from memory, there was extreme evidence for an effect of Object (BF_10_ > 100), and for an interaction between Object and Order (BF_10_ > 100); however, there was also strong evidence for a null effect of Order alone (BF_10_ = 0.05). Bayesian t-tests yielded extreme evidence that the probability of recall was higher when the second target was presented inside the same object with the first, when the memory stimuli and the objects were presented simultaneously (BF_10_ > 100). For memory precision, statistical tests revealed moderate evidence for an effect of Order (BF_10_ = 3.05), and anecdotal evidence for an effect of Object (BF_10_ = 2.5), while no evidence was found for an effect of their interaction. Bayesian t-tests furthermore provided moderate evidence for an effect of Object when the object and memory stimulus were presented simultaneously (BF_10_ = 10).

## Discussion

This study examined whether presenting visual information in an object-based manner improves memory maintenance in VWM. Together the four experiments demonstrated that representations of two visual stimuli were indeed more effectively remembered when they were part of the same object. This benefit was obtained with simple contour-based objects, and whether a pair of memory features were from the same or from different dimensions. Overall memory performance and the object benefit were specifically enhanced for features from orthogonal dimensions; however, this came at the cost of lower memory precision. The object benefit furthermore still emerged when the relative importance of the objects themselves was reduced by presenting them in fixed spatial locations. Finally, it was also confirmed that the object benefit arose automatically, or at least did not depend on strategic use of object information.

### Object benefits in WM

Our findings were consistent with the studies of Xu (2002a, 2002b, 2006), who observed memory benefits for features from different parts of an object in a change detection paradigm. Similarly, in the current study, we tested participants’ VWM for features that were organized as multiple parts of an object, but instead of the same/different probes in the change detection paradigm, we used continuous reproduction of memory features, which allows model-based parameter estimation that provides further insight into the nature of memory representation in VWM. We found that features that were part of the same object had a higher chance of being maintained in memory (i.e., lower guess rate), while the precision of these representations was not improved. As a matter of fact, memory precision decreased when the objects contained non-interfering features (i.e., from different dimensions; Experiment 2). A possible explanation for this reversed effect for memory precision is that non-interfering features might be processed more in parallel (e.g., attentionally; (Krummenacher et al., 2001; Müller et al., 1995; Wheeler & Treisman, 2002; Wolfe et al., 1990), which might facilitate their entry to the memory, such that more information is retained overall. However, this might in turn reduce memory precision, as there is comparatively more information being held in memory.

Furthermore, we found that object-based representation did produce a benefit for the same-dimensional feature, as found originally by Luck and Vogel (1997), but unlike several subsequent change detection studies that failed to find object benefits for same-dimensional feature conjunctions (typically two-color combinations; Delvenne & Bruyer, 2004; Wheeler & Treisman, 2002; Xu, 2002b). It has been proposed that this failure to replicate the object benefit for same-dimensional features could be specific to the change detection task. Awh et al. (2007) used a change detection task where they tested cross-category versus within-category changes between sample and test array. It was assumed that sample-test similarity was higher in within-category change compared to cross-category change, and that this would consequently result in decreasing change detection performance. Indeed, a strong correlation was found between the reduction of memory capacity and sample-test similarity, suggesting that comparing the sample and test may be more difficult for within-category changes, which may cause more confusion in identifying change.

A study by Luria and Vogel (2011) further tested this assumption using the Contralateral Delay Activity (CDA), which is a marker of the number of objects during WM maintenance (e.g., Akyürek et al., 2017; Balaban & Luria, 2015a, 2015b; Luria & Vogel, 2011, 2014; Peterson et al., 2015; Wilson et al., 2012; Woodman & Vogel, 2008). Indeed, Luria and Vogel (2011) found that a small cost was visible in CDA amplitude for a bicolor object, compared to a single-color object during the retention interval, even though no accuracy advantage was found for the former in the behavioral results. This outcome supported the hypothesis that two-color features could be maintained within a single, bound object in VWM.

Since we used a continuous reproduction paradigm, in contrast to a change detection paradigm, our task did not require the comparison/decision process needed to make a comparison between the memory and test arrays. Therefore, the object benefit that occurs before the test phase of the task might also be obtained in behavioral performance, as we indeed observed. Additionally, another important aspect of our study that might have facilitated the object benefit for the same-dimensional features was that the total items given to memorize by the participants was under the typical working memory capacity limit (presumably at least four items; Cowan, 2001; Irwin & Andrews, 1996; Luck & Vogel, 1997; Vogel & Machizawa, 2004). This might have limited overall interference amongst same-dimensional features, and consequently revealed memory advantages for objects. Anecdotal evidence was found that memory precision also decreased in Experiment 4B. Precision also decreased in all other experiments (Table 1 and Table 2 in the Supplementary material show mean parameter estimates for the best fitting model in all experiments), although the effect was not significant enough to be seen as evidence in the individual Bayesian analyses. With that caveat, this potential broader effect might be explained as follows: Possibly, we are seeing two different types of recall: one being recall of the second feature from a discrete memory of the second target, which has a relatively high precision, and the second being recall of the second feature from a memory of the object including the second target, which has a somewhat lower precision. The increased probability of recall of the second feature under in-object conditions then goes hand in hand with decreased precision of second target response as the relative frequency of the second type of recall increases. Future research into this effect and its background could potentially be of benefit to the field.

This account can also explain the larger increase in memory probability for object features that were a combination of color and orientation in Experiment 2, a finding that was consistent with Treisman’s feature integration theory (Treisman & Gelade, 1980), which suggests that less interference should occur when different-dimensional features are maintained in memory. In line with this idea, memory advantages for objects containing a conjunction of different-dimensional features have been found by several studies (Delvenne & Bruyer, 2004; Olson & Jiang, 2002; Riggs et al., 2011; Wheeler & Treisman, 2002).

Other neurophysiological evidence supporting object-based representation in memory comes from functional Magnetic Resonance Imaging (fMRI) data. It has been shown that brain activity in the parietal cortex correlates with object-based representation and grouping of visual elements (Xu & Chun, 2007). These authors described two stages in visual object processing. The first stage was called object individuation and is characterized by attention-related processing. In this stage, a fixed number of objects can be selected, regardless of object complexity. This stage was characterized by a linear increase of neural activity in the inferior intraparietal sulcus (IPS) up to 4 items, after which the neural activity reached a plateau. In the second stage, which was called object identification, objects that were selected in the previous stage are encoded and stored with more detail in VWM. The brain response during this stage was strongest in the superior IPS.

The present results may be related to this two-stage model of object perception, as follows. Since the total number of items (i.e., three) was likely below the maximum capacity of the first object processing stage, they might all be processed in this stage, regardless of whether they were perceived as part of an object, or individually. However, being able to reproduce object-related (featural) information not only requires detecting and attending to the objects, but also successful storage of that information in VWM. Therefore, the increased probability of the second target feature being present in memory when it was part of the object might originate in the second stage of object processing, which is sensitive to object complexity.

It is also worth mentioning that some of our results appear to be compatible with the continuous resource model rather than the discrete slots model of WM. These are the two main models that have been introduced to explain the nature of WM capacity limits. The discrete slots model assumes that working memory storage is limited to a number of discrete slots, typically three or four (Irwin, 1992; Luck & Vogel, 1997; Vogel et al., 2001). In this model, once the amount of items in WM reaches the limit of these slots, no more items can enter memory. Consequently, the discrete slots model suggests that the precision of the memory representation remains constant when the presented memory items exceed the maximum capacity of the slots. On the other hand, the continuous resource model assumes that there is no upper limit of items that can be maintained in working memory, and that memory resources can be flexibly allocated to each item (van den Berg et al., 2012). In this regard, the discrete slots model predicts fixed precision no matter how complex the item is, whereas the continuous resource model predicts more variability in the precision of items. In this study we found object effects on the probability of having the second target in memory. However, we also found some evidence that precision differed between object conditions in Experiment 2 and Experiment 4a, which may suggest that there is some variability in the precision of the encoded items in memory.

### Attentional effects

Although both the number of features and objects were presumably well under the maximum capacity of VWM, recall accuracy was clearly different between the two targets regardless of object conditions: Accuracy on the second target was always (much) lower than that of the first. This was expected, firstly because the first target feature was also the one to be tested first after the memory display. Secondly, in our experimental design, the first target feature was always flagged as such, because it appeared in red or within a colored circle, to ensure this part of the object was always encoded. This made the first target feature salient, and likely to draw the focus of attention. The first target should therefore have perceptual priority in the encoding stage of VWM. Indeed, we hypothesized that such prioritization may facilitate the rest of the object as well and result in better memory for the second target, when presented within the same object with the first target.

The study of Egly et al. (1994) suggested that when attention is drawn towards one part of the object, it can spread within boundary of object, therefore the rest of the attended object can be selected automatically. Furthermore, it has been argued that perceiving individual parts as an integrated object depends on where attention is focused exactly, and whether this includes the object structure (Driver & Baylis, 1998; Marr, 1982). Our finding is consistent with such an object-based attention account. It must be noted that in our study, participants were only required to memorize the individual features of orientation or color, so the simple background shapes (i.e., the objects) that encompassed these memory stimuli were not task relevant. The object was also never predictive of the second feature to be tested. It could be expected that participants might, thus, only attend to the task-relevant parts of the object rather than on the object as whole. Moreover, by presenting the task-relevant parts of the object in fixed locations in Experiment 3, participants might even be able to attend more to the locations of the features themselves, rather than to the object itself. Nevertheless, even under these conditions that rendered the object itself completely task-irrelevant, the features in all of our experiments were still perceived as part of a bound object, as indicated by the presence of the object effect in each experiment. This finding suggests that the objects were processed at an early stage of visual processing, possibly reliant on automatic perceptual grouping (Driver et al., 2001; Duncan, 1984; Duncan & Humphreys, 1989; Kahneman & Treisman, 1984).

Conversely, a recent study by Chen et al. (2021) investigated perceptual grouping benefits for features that were either grouping-relevant, or not. While grouping-relevant features produced clear benefits, grouping-irrelevant features did not, unless both feature and grouping were task relevant. The authors concluded that features may be encoded independently in VWM, and integrated object representations are not automatically generated, but instead depend on the task demands. This would seem to be at odds with the object benefits that were presently observed, given that our objects were always task irrelevant. However, the highlighted first feature used in our study may actually have put the object as a whole into focus, as argued above, thereby ‘activating’ its benefits.

Finally, the current results seems inconsistent with the view that attentional prioritization of WM items can occur before or after the appearance of object features, even though the first target feature clearly benefitted from prioritization due to its unique color. Previous studies that showed pre- or retro-cue benefits on memory (Bays & Husain, 2008; Schmidt et al., 2002) used those cues to draw attention to certain memory items indicating that they are more likely to be tested. Therefore, the cued item in these studies was directly task-relevant information essential to be recalled. In Experiment 4 of our study, the features that needed to be memorized were always presented together in the same display, and the object shapes that made them part of the same or of a different object either preceded or followed the memory features. Therefore, the part of the object that was presented before or after the memory features was not directly task-relevant information that participants needed to retain in memory, and this object information did not necessarily need to be used. Thereby, any encoding of this shape and any benefit it might bring would be purely strategic in nature. We found that participants were unable to use the object shape strategically, to help structure VWM contents either during encoding or maintenance, depending on whether the object preceded or followed the features. The object effects we presently observed, thus, seemed to be driven by processing stages that precede the strategic level. However, this finding does not imply that memory benefits for objects can emerge only with simultaneous perception of visual information, or that it is limited to the perceptual/encoding stage in all cases. For instance, one previous study found that the presentation of objects based on various Gestalt principles (collinearity, closure, and similarity) across two sequential stimulus displays improved VWM performance (Gao et al., 2016). In the current study, the complete task-irrelevance of the object might have led to it not being selected attentionally, thereby precluding any positive effect.

To conclude, in four experiments we presented consistent evidence demonstrating that object-based presentation of visual information helped our participants to retain more information in VWM, even when the total number of items was well below the VWM capacity limit. Recall advantages were obtained when combining features from either the same or different dimensions into a single object. The object benefit seemed to happen automatically at a relatively early stage of visual processing, which was indicated by the persistent effect of the object even when participants' attention was made to focus more on the location of the features rather than on the object surrounding the stimuli, and by a lack of evidence for strategic encoding or maintenance.

## Supplementary Information

Below is the link to the electronic supplementary material.

Supplementary file1 (DOCX 251 KB)

## Data Availability

The datasets and analysis scripts that were used in this study are available in the Open Science Framework repository, with the identifier xqk3w (https://osf.io/xqk3w/; https://doi.org/10.17605/OSF.IO/XQK3W).

## References

[CR1] Akyürek EG, Kappelmann N, Volkert M, van Rijn H (2017). What you see is what you remember: Visual chunking by temporal integration enhances working memory. Journal of Cognitive Neuroscience.

[CR2] Allport DA (1971). Parallel encoding within and between elementary stimulus dimensions. Perception & Psychophysics.

[CR3] Awh E, Barton B, Vogel EK (2007). Visual working memory represents a fixed number of items regardless of complexity. Psychological Science.

[CR4] Baddeley A (2003). Working memory: Looking back and looking forward. Nature Reviews Neuroscience.

[CR5] Balaban H, Luria R (2015). Integration of distinct objects in visual working memory depends on strong objecthood cues even for different-dimension conjunctions. Cerebral Cortex.

[CR6] Balaban H, Luria R (2015). The number of objects determines visual working memory capacity allocation for complex items. NeuroImage.

[CR7] Barnes LL, Nelson JK, Reuter-Lorenz PA (2001). Object-based attention and object working memory: Overlapping processes revealed by selective interference effects in humans. Progress in Brain Research.

[CR8] Bays PM, Husain M (2008). Dynamic shifts of limited working memory resources in human vision. Science.

[CR9] Brady TF, Konkle T, Alvarez GA (2011). A review of visual memory capacity: Beyond individual items and toward structured representations. Journal of Vision.

[CR10] Brainard DH (1997). The Psychophysics Toolbox. Spatial Vision.

[CR11] Chen S, Kocsis A, Liesefeld HR, Müller HJ, Conci M (2021). Object-based grouping benefits without integrated feature representations in visual working memory. Attention, Perception, & Psychophysics.

[CR12] Chen Z, Cowan N (2005). Chunk limits and length limits in immediate recall: A reconciliation. Journal of Experimental Psychology: Learning, Memory, and Cognition.

[CR13] Cowan N (2001). The magical number 4 in short-term memory: A reconsideration of mental storage capacity. Behavioral and Brain Sciences.

[CR14] Cowan N, Izawa C, Ohta N (2005). Working memory capacity in a theoretical context. Human learning and memory: Advances in theory and application.

[CR15] Daneman M, Carpenter PA (1980). Individual differences in working memory and reading. Journal of Verbal Learning and Verbal Behavior.

[CR16] Dell Acqua R, Sessa P, Toffanin P, Luria R, Jolicoeur P (2010). Orienting attention to objects in visual short-term memory. Neuropsychologia.

[CR17] Delvenne J-F, Bruyer R (2004). Does visual short-term memory store bound features?. Visual Cognition.

[CR18] Driver J, Baylis GC, Parasuraman R (1998). Attention and visual object segmentation. The attentive brain.

[CR19] Driver J, Davis G, Russell C, Turatto M, Freeman E (2001). Segmentation, attention and phenomenal visual objects. Cognition.

[CR20] Duncan J (1984). Selective attention and the organization of visual information. Journal of Experimental Psychology: General.

[CR21] Duncan J, Humphreys GW (1989). Visual search and stimulus similarity. Psychological Review.

[CR22] Egly R, Driver J, Rafal RD (1994). Shifting visual attention between objects and locations: Evidence from normal and parietal lesion subjects. Journal of Experimental Psychology: General.

[CR23] Eimer M, Kiss M (2010). An electrophysiological measure of access to representations in visual working memory. Psychophysiology.

[CR24] Elsley JV, Parmentier FB (2015). The asymmetry and temporal dynamics of incidental letter location bindings in working memory. The Quarterly Journal of Experimental Psychology.

[CR25] Fukuda K, Vogel E, Mayr U, Awh E (2010). Quantity, not quality: The relation between fluid intelligence and working memory capacity. Psychonomic Bulletin & Review.

[CR26] Gao Z, Gao Q, Tang N, Shui R, Shen M (2016). Organization principles in visual working memory: Evidence from sequential stimulus display. Cognition.

[CR27] Gao Z, Li J, Yin J, Shen M (2010). Dissociated mechanisms of extracting perceptual information into visual working memory. PLoS One.

[CR28] Gelman A, Hwang J, Vehtari A (2014). Understanding predictive information criteria for Bayesian models. Statistics and Computing.

[CR29] Gobet F, Lane P, Croker S, Cheng P, Jones G, Oliver I, Pine J (2001). Chunking mechanisms in human learning. Trends in Cognitive Sciences.

[CR30] Griffin IC, Nobre AC (2003). Orienting attention to locations in internal representations. Journal of Cognitive Neuroscience.

[CR31] Hardman KO, Vergauwe E, Ricker TJ (2017). Categorical working memory representations are used in delayed estimation of continuous colors. Journal of Experimental Psychology: Human Perception and Performance.

[CR32] Hollingworth A (2007). Object-position binding in visual memory for natural scenes and object arrays. Journal of Experimental Psychology: Human Perception and Performance.

[CR87] Irwin, D. E. (1992). Memory for position and identity across eye movements. *Journal of Experimental Psychology: Learning, Memory, and Cognition,**18*(2), 307–317. 10.1037/0278-7393.18.2.307

[CR33] Irwin DE, Andrews R, Inui T, McClelland J (1996). Integration and accumulation of information across saccadic eye movements. Attention and performance. XVI.

[CR88] JASP Team (2022). JASP (Version 0.16.3)[Computer software]. https://jasp-stats.org/

[CR34] Jonides J, Smith EE, Koeppe RA, Awh E, Minoshima S, Mintun MA (1993). Spatial working memory in humans as revealed by PET. Nature.

[CR35] Kahneman D, Treisman A, Parasuraman R, Davis DR (1984). Changing views of attention and automaticity. Varieties of attention.

[CR36] Kleiner, M., Brainard, D., & Pelli, D. (2007). What’s new in psychtoolbox-3. *Perception, 36*, ECVP Abstract Supplement.

[CR37] Krummenacher J, Müller HJ, Heller D (2001). Visual search for dimensionally redundant pop-out targets: Evidence for parallel-coactive processing of dimensions. Perception & Psychophysics.

[CR38] Kuo B-C, Rao A, Lepsien J, Nobre AC (2009). Searching for targets within the spatial layout of visual short-term memory. Journal of Neuroscience.

[CR39] Landman R, Spekreijse H, Lamme VAF (2003). Large capacity storage of integrated objects before change blindness. Vision Research.

[CR40] Luck SJ, Vogel EK (1997). The capacity of visual working memory for features and conjunctions. Nature.

[CR41] Luria R, Vogel EK (2011). Shape and color conjunction stimuli are represented as bound objects in visual working memory. Neuropsychologia.

[CR42] Luria R, Vogel EK (2014). Come together, right now: Dynamic overwriting of an object's history through common fate. Journal of Cognitive Neuroscience.

[CR43] Makovski T, Sussman R, Jiang YV (2008). Orienting attention in visual working memory reduces interference from memory probes. Journal of Experimental Psychology: Learning, Memory, and Cognition.

[CR44] Marr D (1982). Vision.

[CR45] Matsukura M, Luck SJ, Vecera SP (2007). Attention effects during visual short-term memory maintenance: Protection or prioritization?. Attention, Perception, & Psychophysics.

[CR46] Matsukura M, Vecera SP (2009). Interference between object-based attention and object-based memory. Psychonomic Bulletin and Review.

[CR47] McCarthy G, Blamire AM, Puce A, Nobre AC, Bloch G, Hyder F, Goldman-Rakic P, Shulman RG (1994). Functional magnetic resonance imaging of human prefrontal cortex activation during a spatial working memory task. Proceedings of the National Academy of Sciences of the United States of America.

[CR48] Miller GA (1956). The magical number seven plus or minus two: Some limits on our capacity for processing information. Psychological Review.

[CR49] Miyake A, Friedman NP, Rettinger DA, Shah P, Hegarty M (2001). How are visuospatial working memory, executive functioning and spatial abilities related? A latent variable analysis. Journal of Experimental Psychology: General.

[CR50] Müller HJ, Heller D, Ziegler J (1995). Visual search for singleton feature targets within and across feature dimensions. Perception & Psychophysics.

[CR51] Olson IR, Jiang Y (2002). Is visual short-term memory object based? Rejection of the “strong-object” hypothesis. Perception & Psychophysics.

[CR52] Olson IR, Marshuetz C (2005). Remembering what brings along where in visual working memory. Perception & Psychophysics.

[CR53] Parra MA, Cubelli R, Della Sala S (2011). Lack of color integration in visual short-term memory binding. Memory & Cognition.

[CR54] Pelli DG (1997). The Video Toolbox software for visual psychophysics: Transforming numbers into movies. Spatial Vision.

[CR55] Pertzov Y, Bays PM, Joseph S, Husain M (2013). Rapid forgetting prevented by retrospective attention cues. Journal of Experimental Psychology: Human Perception and Performance.

[CR56] Peterson DJ, Gözenman F, Arciniega H, Berryhill ME (2015). Contralateral delay activity tracks the influence of gestalt grouping principles on active visual working memory representations. Attention, Perception, & Psychophysics.

[CR57] Ravizza SM, Uitvlugt MG, Hazeltine E (2016). Where to start? Bottom-up attention improves working memory by determining encoding order. Journal of Experimental Psychology: Human Perception and Performance.

[CR58] Rensink RA (2000). Seeing, sensing, and scrutinizing. Vision Research.

[CR85] Ricker, T. J., & Hardman, K. O. (2017). The nature of short-term consolidation in visual working memory. *Journal of Experimental Psychology: General,**146*(11), 1551–1573. 10.1037/xge0000346 10.1037/xge0000346 28703619

[CR59] Riggs KJ, Simpson A, Potts T (2011). The development of visual short-term memory for multifeature items during middle childhood. Journal of Experimental Child Psychology.

[CR60] Saiki J (2016). Location-unbound color-shape binding representations in visual working memory. Psychological Science.

[CR61] Schmidt BK, Vogel EK, Woodman GF, Luck SJ (2002). Voluntary and automatic attentional control of visual working memory. Perception & Psychophysics.

[CR62] Schneegans S, Bays PM (2017). Neural architecture for feature binding in visual working memory. Journal of Neuroscience.

[CR63] Souza AS, Oberauer K (2016). In search of the focus of attention in working memory: 13 Years of the retro-cue effect. Attention, Perception, & Psychophysics.

[CR64] Suchow JW, Brady TF, Fougnie D, Alvarez GA (2013). Modeling visual working memory with the MemToolbox. Journal of Vision.

[CR65] Treisman AM, Gelade G (1980). A feature-integration theory of attention. Cognitive Psychology.

[CR66] Treisman A, Zhang W (2006). Location and binding in visual working memory. Memory & Cognition.

[CR67] Udale R, Farrell S, Kent C (2017). No evidence for binding of items to task-irrelevant backgrounds in visual working memory. Memory & Cognition.

[CR68] Van den Berg R, Shin H, Chou W-C, George R, Ma WJ (2012). Variability in encoding precision accounts for visual short-term memory limitations. Proceedings of the National Academy of Sciences of the United States of America.

[CR69] Vogel EK, Machizawa M (2004). Neural activity predicts individual differences in visual working memory capacity. Nature.

[CR70] Vogel EK, Woodman GF, Luck SJ (2001). Storage of features, conjunctions, and objects in visual working memory. Journal of Experimental Psychology: Human Perception and Performance.

[CR71] Wang B, Cao X, Theeuwes J, Olivers CNL, Wang Z (2016). Location-based effects underlie feature conjunction benefits in visual working memory. Journal of Vision.

[CR72] Wang B, Cao X, Theeuwes J, Olivers CNL, Wang Z (2017). Separate capacities for storing different features in visual working memory. Journal of Experimental Psychology: Learning, Memory, and Cognition.

[CR86] Westfall, P. H., Johnson, W. O., UTTS, J M. (1997). A Bayesian perspective on the Bonferroni adjustment. *Biometrika,**84*(2), 419–427. 10.1093/biomet/84.2.419

[CR84] Wetzels, R., Matzke, D., Lee, M. D., Rouder, J. N., Iverson, G. J., & Wagenmakers, E.-J. (2011). Statistical Evidence in Experimental Psychology: An Empirical Comparison Using 855 t Tests. *Perspectives on Psychological Science,**6*(3), 291–298. 10.1177/1745691611406923 10.1177/1745691611406923 26168519

[CR73] Wheeler ME, Treisman AM (2002). Binding in short-term visual memory. Journal of Experimental Psychology: General.

[CR74] Wickham H (2016). Ggplot2: Elegant graphics for data analysis.

[CR75] Wilson KE, Adamo M, Barense MD, Ferber S (2012). To bind or not to bind: Addressing the question of object representation in visual short-term memory. Journal of Vision.

[CR76] Wolfe JM, Yu KP, Stewart MI, Shorter AD, Friedman-Hill SR, Cave KR (1990). Limitations on the parallel guidance of visual search: Color × Color and Orientation × Orientation conjunctions. Journal of Experimental Psychology: Human Perception and Performance.

[CR77] Woodman GF, Vogel EK (2008). Selective storage and maintenance of an object's features in visual working memory. Psychonomic Bulletin & Review.

[CR78] Xu Y (2002). Encoding color and shape from different parts of an object in visual short-term memory. Perception & Psychophysics.

[CR79] Xu Y (2002). Limitations of object-based feature encoding in visual short-term memory. Journal of Experimental Psychology: Human Perception and Performance.

[CR80] Xu Y (2006). Understanding the object benefit in visual short-term memory: The roles of feature proximity and connectedness. Perception & Psychophysics.

[CR81] Xu Y, Chun MM (2007). Visual grouping in human parietal cortex. Proceedings of the National Academy of Sciences of the United States of America.

[CR82] Yin J, Zhou J, Xu H, Liang J, Gao Z, Shen M (2012). Does high memory load kick task-irrelevant information out of visual working memory?. Psychonomic Bulletin & Review.

[CR83] Zhang W, Luck SJ (2008). Discrete fixed-resolution representations in visual working memory. Nature.

