# Supplementary material for: Object-based visual working memory: an object benefit for equidistant memory items presented within simple contours
Source: Psychol Res. 2022 Oct 29;87(5):1569–89. doi: 10.1007/s00426-022-01757-w (PMC10227135; doi:10.1007/s00426-022-01757-w)
Supplement: Supplementary file 1 — Supplementary file1 (DOCX 251 KB) [file 426_2022_1757_MOESM1_ESM.docx]

**Supplementary Material**

**Analysis for the switching cost of the target locations in Experiment 3**

To explore whether there was a cost for the switch of the target position in Experiment 3we first computed the average of each fixed location for both object conditions and both target responses separately (Figure 1). A Bayesian ANOVA was used to test main effect of fixed location, object condition and their interactions. For first targets, there were no evidence for main effect of fixed location, object or their interaction. For the second response, there was substantial evidence for a main effect of object (BF_inc-10_ = 4.33), but no evidence for main effect of fixed location or their interaction.


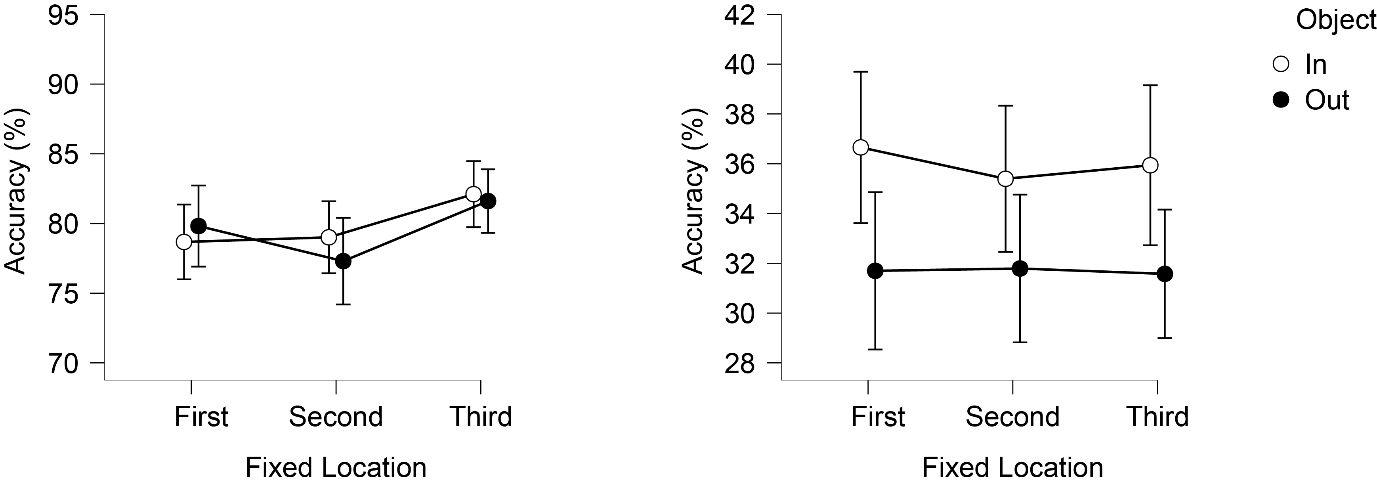


**Fig. 1** Average accuracy of each fixed location for both object conditions and first target (left) and second target (right) locations. Error bars in figures represent 95% confidence intervals.

Furthermore, assuming that switching costs might happen mostly at the beginning of the block, we divided each of the three blocks into two halves, and computed average accuracy of each half for each fixed location and two object conditions separately (Figure 2). Bayesian ANOVA revealed substantial evidence for main effect of two halves (BF_inc-10_ = 4.07) and anecdotal evidence for an interaction of two halves and fixed locations (BF_inc-10_ = 1.72) for the first response. Post hoc tests revealed substantial evidence for a significant difference between the two halves of the first fixed location (BF_10_ = 3.23), there was anecdotal evidence for significant difference between the two halves of the third fixed location (BF_10_ = 2.35). For the second response, there was substantial evidence for a main effect of object (BF_inc-10_ = 6.71) while there was no evidence for other main effects or interactions. Switching costs cannot apply for the first location, so we consequently attribute the first target effect to the participants getting used to the experiment. As no effect was found for the second location (first half accuracy is even slightly better than second half accuracy) and only anecdotal evidence is found for an effect for the third location we conclude that no significant switching costs occurred.


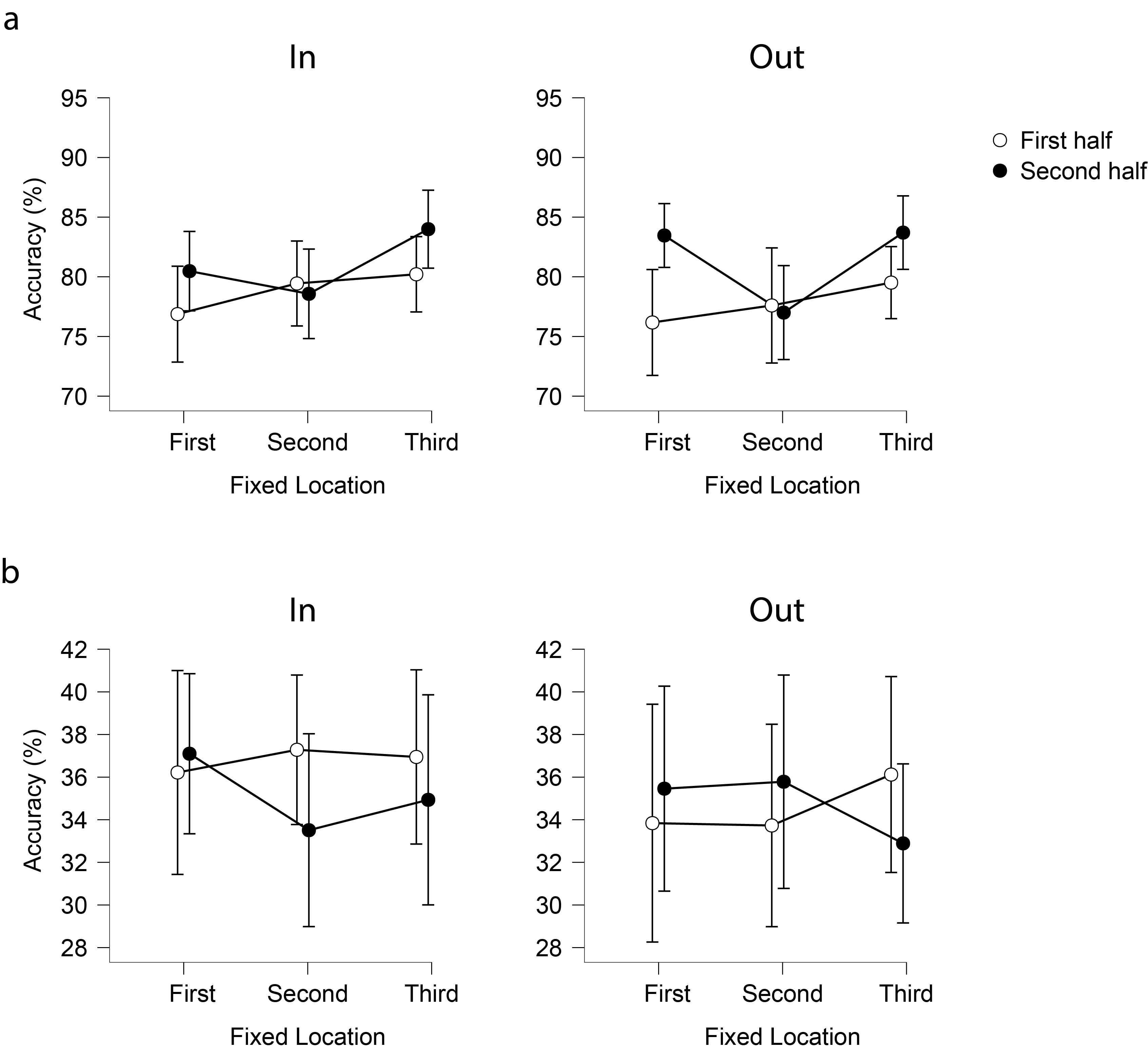


**Fig. 2** Average accuracy of each fixed location for two halfs are shown for both conditions seperatly. Panel a shows first target accuracy and panel b shows second target accuracy. Error bars in figures represent 95% confidence intervals.

**Parameter estimates from the best fitting model across conditions and participants**

**Table 1.** Mean Parameter Estimates for the Best-Fitting Model in Experiment 1-3

|  | | **T1** | | **T2** | |
| --- | --- | --- | --- | --- | --- |
|  |  | ***P^M^*** | **σ (°)** | ***P^M^*** | **σ (°)** |
| Exp. 1 | In | 0.94 | 11.96 | 0.43 | 19.19 |
|  | Out | 0.94 | 11.96 | 0.22 | 18.16 |
| Exp. 2 | In | 0.94 | 8.95 | 0.79 | 26.54 |
|  | Out | 0.94 | 8.95 | 0.46 | 22.15 |
| Exp. 3 | In | 0.88 | 13.36 | 0.36 | 17.67 |
|  | Out | 0.88 | 13.36 | 0.24 | 16.60 |
|  |  |  |  |  |  |

Note. Exp = experiment; ***P^M^*** = the probability an item is in memory; **σ (°) =** standard deviation of the response; In = T2 was inside of the object containing T1; Out = T2 was outside of the object containing T1.

**Table 2.** Mean Parameter Estimates for the Best-Fitting Model in Experiment 4a-4b

|  |  | | **T1** | | **T2** | |
| --- | --- | --- | --- | --- | --- | --- |
|  |  |  | ***P^M^*** | **σ (°)** | ***P^M^*** | **σ (°)** |
| Exp. 4a | Before | In | 0.96 | 11.25 | 0.41 | 16.53 |
|  |  | Out | 0.96 | 11.25 | 0.42 | 16.40 |
|  | Simultaneous | In | 0.94 | 12.18 | 0.48 | 18.54 |
|  |  | Out | 0.94 | 12.18 | 0.28 | 16.55 |
|  | After | In | 0.93 | 12.13 | 0.41 | 17.31 |
|  |  | Out | 0.93 | 12.13 | 0.41 | 18.35 |
| Exp. 4b | Before | In | 0.93 | 11.69 | 0.33 | 18.05 |
|  |  | Out | 0.93 | 11.69 | 0.37 | 18.20 |
|  | Simultaneous | In | 0.91 | 12.43 | 0.46 | 20.17 |
|  |  | Out | 0.91 | 12.43 | 0.14 | 15.21 |
|  | After | In | 0.89 | 11.89 | 0.31 | 16.17 |
|  |  | Out | 0.89 | 11.89 | 0.30 | 15.21 |
|  |  |  |  |  |  |  |

Note. Exp = experiment; ***P^M^*** = the probability an item is in memory; **σ (°) =** standard deviation of the response; In = T2 was inside of the object containing T1; Out = T2 was outside of the object containing T1; Before = the object shape appears before the gratings; Simultaneous = the object shape appears simultaneous with the gratings; After = the object shape appears after the gratings.
